# Neoadjuvant chemoradiotherapy with camrelizumab in patients with locally advanced esophageal squamous cell carcinoma

**DOI:** 10.3389/fsurg.2022.893372

**Published:** 2022-08-02

**Authors:** Fei Chen, Lingdong Qiu, Yushu Mu, Shibin Sun, Yulong Yuan, Pan Shang, Bo Ji, Qifei Wang

**Affiliations:** ^1^ Department of Gastroenterology, The Second Affiliated Hospital of Shandong First Medical University, Taian, China.; ^2^ Department of Thoracic Surgery, The Second Affiliated Hospital of Shandong First Medical University, Taian, China.

**Keywords:** esophageal squamous cell carcinoma, neoadjuvant therapy, camrelizumab, chemoradiotherapy, survival

## Abstract

**Background:**

Neoadjuvant anti-programmed death receptor-1 (PD-1) blockade has been reported to improve the prognosis of locally advanced esophageal squamous cell carcinoma (ESCC). This study was aimed to evaluate the efficacy and safety of neoadjuvant camrelizumab plus chemoradiotherapy in locally advanced ESCC.

**Methods:**

We retrospectively enrolled ESCC patients who received camrelizumab plus chemoradiotherapy as neoadjuvant therapy before surgery from May 2019 to September 2021.

**Results:**

A total of 38 eligible patients were enrolled. The neoadjuvant treatment was well tolerated with no serious treatment-related adverse events. 36 (94.7%) patients achieved a R0 resection without hospital mortality or any other serious intraoperative complications. The objective response rate (ORR) was 63.2% and the disease control rate (DCR) was 100.0%. The major pathological response (MPR) was 50.0% and the complete pathological response (pCR) was 39.5%. With a median follow-up of 18.5 months, 6 (15.8%) patients had died. The overall survival (OS) and disease-free survival (DFS) at 12 months were 87.6% and 78.7%, respectively. Subgroup analysis demonstrated that patients who got MPR or pCR achieved improved survival, while PD-L1 expression did not reach statistically difference in predicting survival.

**Conclusions:**

Neoadjuvant camrelizumab plus chemoradiotherapy is safe and efficacious in treating patients with locally advanced ESCC.

## Introduction

With over 500,000 newly diagnosed cases and 509,000 annual deaths, esophageal carcinoma ranks as the seventh most common cancer and the sixth leading cause of cancer death worldwide in 2018 (1). Esophageal squamous cell carcinoma (ESCC) is the predominant histologic subtype, accounting for 87% of all esophageal cancers (2). Surgery remains as the mainstay treatment for patients with early-stage ESCC; however, a great proportion of patients shave developed into locally advanced stage, and surgery alone is not satisfactory due to high recurrence and metastasis rate (3). With the advent of neoadjuvant therapy, preoperative chemoradiotherapy followed by surgery has developed into the standard-of-care treatment for locally advanced ESCC (4–6).

Recently, anti-programmed death 1 (PD-1) antibodies have paved the way for a new era of cancer immunotherapy, and thus have been approved by the Food and Drug Administration as second-line treatment in unresectable ESCC patients (7, 8). Neoadjuvant administration of anti-PD-1 agents has demonstrated encouraging efficacy with favorable tolerability in other malignancies including lung cancer, melanoma and colorectal cancer (9–11). Moreover, anti-PD-1 agents combined with chemotherapy or chemoradiation has been exploited in a neoadjuvant treatment setting for locally advanced ESCC, the results of which produced an acceptable therapeutic response and a low-toxicity profile (12–15). For example, Park et al. reported that neoadjuvant pembrolizumab plus platinum-based chemoradiotherapy may not increase the operative risk or reduce the quality of radical dissection including lymphadenectomy (16).

Notably, camrelizumab, a novel IgG4-kappa PD-1 inhibitor developed in China, has been previously witnessed for adjuvant or second-line therapy of locally advanced ESCC (17, 18). However, the research on neoadjuvant camrelizumab plus chemoradiotherapy in treating locally advanced ESCC is limited. This retrospective study was aimed to evaluate the efficacy and safety of camrelizumab combined with chemoradiotherapy in neoadjuvant treatment of locally advanced ESCC.

## Materials and methods

### Patient selection

From May 2019 to September 2021, we retrospectively recruited ESCC patients who received camrelizumab plus chemoradiotherapy as neoadjuvant therapy before surgery at the Second Affiliated Hospital of Shandong First Medical University (Shandong, China). The key inclusion criteria were the following: (1) Patients with histologically confirmed locally advanced ESCC, which was defined as cT1N1-3M0 or cT2-4aN0-3M0 (AJCC, 8th Edition); (2) Patients were aged 18 years or older; (3) Eastern Cooperative Oncology Group (ECOG) performance status (PS) of 0 or 1; and (4) With adequate organ function for surgical resection. Patients were excluded when they had other anti-tumor treatments before or during the neoadjuvant treatment, immunodeficiency disease, and other significant concurrent malignant tumors. The study was approved by institutional review board of Second Affiliated Hospital of Shandong First Medical University, and carried out in accordance with the Helsinki Declaration (as revised in 2013). Written informed consent was obtained from all patients.

### Neoadjuvant therapy and surgical procedures

Camrelizumab was given 200 mg intravenously every 3 weeks (a cycle). Simultaneously, the paclitaxel was administered intravenously at a dose of 100 mg/m^2^ of body-surface area on days 1 and 8, and the carboplatin was administered intravenously at an area under the curve of 5 mg/ml per minute on day 1. The intensity-modulated radiotherapy was given according to Chinese treatment guidelines for esophageal carcinoma (19), and was prescribed to cover 95% of the planning gross tumor volume (PGTV), given at 2.0 Gy per fraction, five fractions per week. The surgery was performed at the surgeon's decision after completion of at least 2 cycles of neoadjuvant therapy. Patients were re-evaluated with contrast-enhanced CT within 1 week before surgery. Standard minimally invasive esophagectomy (MIE) was performed for all patients, and the upper tumor mainly was treated with three-incision McKeown surgery (three fields or two fields). The middle and lower segment tumors with two-incision Ivor-Lewis surgery. Besides, a gastric tube was applied to reconstruct the digestive tract after esophagectomy. The surgical indicators, including operative time, blood loss, blood transfusion, hospital stay, and resection margin were first recorded. The postoperative complications, including pneumonia, chylothorax, pleural effusion, wound infection, and recurrent nerve paralysis were also recorded. Patients were followed postoperatively with routine CT scans every 3 months in the first year following the treatment, and every 6 months thereafter.

### Assessment

The primary outcome was overall survival (OS) and disease-free survival (DFS) at 12 months. OS was defined as the time from neoadjuvant treatment to date of death, and DFS was defined as the time from neoadjuvant treatment until disease recurrence or death. Secondary outcomes were radiologic response prior to surgery, pathological responses including major pathological response (MPR) and complete pathological response (pCR), and treatment-related adverse events (TRAEs). Radiologic responses were assessed according to Response Evaluation Criteria in Solid Tumors (RECIST) version 1.1. MPR was defined as residual tumor cells ≤10% at the time of surgery, and pCR was defined as tumors without any viable tumor cells. TRAEs were graded according to the National Cancer Institute Common Terminology Criteria for Adverse Events, version 5.0. PD-L1 expression was assessed using the PD-L1 IHC 22C3 pharmDx assay (Agilent Technologies, CA, USA) and was expressed as combined positive score (CPS) by dividing the number of PD-L1-stained tumor and immune cells with the total number of viable tumor cells and multiplying by 100. The tumor PD-L1 expression was defined positive when the CPS ≥1%.

### Statistical analyses

All statistical analyses were done using SPSS 20.0 (IBM SPSS Inc., Chicago, IL, USA). Continuous variables were presented with as a mean ± standard deviation when the normality was verified by Shapiron-Wilk test (*P *> 0.1), otherwise median and range. The categorical variables were expressed as counts and percentages. The Kaplan-Meier method was used to estimate DFS and OS and corresponding 95% CIs. Differences were considered to be significant when *P *< 0.05.

## Results

### Patient characteristics

As shown in Table 1, a total of 38 patients with ESCC were included (31 males, 7 females; median age 59 years). 30 (78.9%) patients had ECOG PS 0 and 8 (21.1%) had ECOG PS 1. More than half of the patients (60.5%) were smokers. Tumors were located in the proximal third of the esophagus in 8 (21.1%) patients, the middle third in 16 (42.1%) patients, and the distal third in 14 (36.8%) patients. There were 7 (18.4%) patients at TNM stage II, 27 (71.1%) patients at stage III, and 4 (10.5%) patients at stage IVA. Moreover, 22 (57.9%) patients had negative PD-L1 expression and 12 (31.6%) patients had positive PD-L1 expression. Time from completion of neoadjuvant therapy to surgery was 31 days (18–46).

**Table 1:** Patient characteristics.

Characteristics	Patients (*n* = 38)
Age, years (mean ± SD)	60.2 ± 5.8
Sex	
Male	31 (81.6%)
Female	7 (18.4%)
ECOG PS	
0	30 (78.9%)
1	8 (21.1%)
Smoking status	
Former or current	23 (60.5%)
Never	15 (39.5%)
Tumor location	
Proximal third	8 (21.1%)
Middle third	16 (42.1%)
Distal third	14 (36.8%)
Histologic grade	
Well	9 (23.7%)
Moderate	15 (39.5%)
Poor	14 (36.8%)
Clinical T stage	
2/3/4a	4 (10.5%)/30 (78.9%)/4 (10.5%)
Clinical N stage	
0/1/2/3	4 (10.5%)/20 (52.6%)/13 (34.2%)/1 (2.6%)
Clinical TNM stage (AJCC, 8th Edition)	
II/III/IVA	7 (18.4%)/27 (71.1%)/4 (10.5%)
PD-L1 status	
Negative	22 (57.9%)
Positive	12 (31.6%)
Unknown	4 (10.5%)
Time from completion of neoadjuvant chemoradiotherapy with camrelizumab to surgery, days, median (range)	31 (18–46)

Abbreviations: ECOG PS, Eastern Cooperative Oncology Group performance status; AJCC, American Joint Committee on Cancer; PD-L1, programmed death-ligand 1.

### Safety

The TRAEs during neoadjuvant treatment are shown in the Table 2. A total of 34 (89.5%) patients experienced some form of TRAEs. Grade 3 adverse events occurred in 8 (21.1%) patients, and no grade 4 or 5 adverse events were observed. Grade 3 radiation esophagitis occurred in 3 (7.9%) patients. Other grade 3 adverse events included pain in 4 (10.5%) patients, decreased WBC count in 2 (5.3%) patients, and fatigue in 1 (2.6%) patient. No treatment-related deaths were reported. Immune-related adverse events occurred in 18 (47.4%) patients. The most common immune-related adverse events were reactive capillary endothelial proliferation in 17 (44.7%) patients, and hypothyroidism in 6 (15.8%) patients, all in grade 1. Other immune-related adverse events including myocarditis, hepatitis, and nephritis were not observed.

**Table 2:** Treatment-related adverse events during neoadjuvant treatment (*n* = 38).

	All	Grade 1	Grade 2	Grade 3	Grade 4
Any treatment-related adverse event	34 (89.5%)	6 (15.8%)	12 (31.6%)	8 (21.1%)	–
Pain	31 (81.6%)	20 (52.6%)	7 (18.4%)	4 (10.5%)	–
Decreased appetite	28 (73.7%)	22 (57.9%)	6 (15.8%)	–	–
WBC count decreased	26 (68.4%)	20 (52.6%)	4 (10.5%)	2 (5.3%)	–
Anemia	25 (65.8%)	22 (57.9%)	3 (7.9%)	–	–
Decreased albumin	25 (65.8%)	19 (50.0%)	6 (15.8%)	–	–
Radiation esophagitis	22 (57.9%)	16 (42.1%)	3 (7.9%)	3 (7.9%)	–
Fatigue	20 (52.6%)	17 (44.7%)	2 (5.3%)	1 (2.6%)	–
Weight loss	20 (52.6%)	20 (52.6%)	–	–	–
Reactive capillary endothelial proliferation	17 (44.7%)	17 (44.7%)	–	–	–
Thrombocytopenia	13 (34.2%)	12 (31.6%)	1 (2.6%)	–	–
Increased ALT/AST	12 (31.6%)	12 (31.6%)	–	–	–
Increased bilirubin	12 (34.2%)	12 (34.2%)	–	–	–
Nausea or vomiting	10 (26.3%)	10 (26.3%)	–	–	–
Diarrhea	8 (21.1%)	7 (18.4%)	1 (2.6%)	–	–
Constipation	8 (21.1%)	8 (21.1%)	–	–	–
Cough	8 (21.1%)	7 (18.4%)	1 (2.6%)	–	–
Radiation dermatitis	6 (15.8%)	5 (13.2%)	1 (2.6%)		
Radiation pneumonia	6 (15.8%)	5 (13.2%)	1 (2.6%)	–	–
Hypothyroidism	6 (15.8%)	6 (15.8%)	–	–	–

### Surgical treatment

The surgical details are presented in the Table 3. 16 (42.1%) patients had the McKewon surgery and 22 (57.9%) patients had Ivor Lewis surgery. The median operation time was 315 min (296–419), the median amount of blood loss was 196 mL (134–431), and the median hospital stay was 16 days (12–24). 36 (94.7%) patients achieved a R0 resection and 2 (5.3%) patients had a R1 resection. During the postoperative periods, 6 (15.8%) patients had pneumonia, 5 (13.2%) had chylothorax, 4 (10.5%) had pleural effusion, 2 (5.3%) had wound infection, and 2 (5.3%) had recurrent nerve paralysis. There was no death in hospital or any other serious intraoperative complications.

**Table 3:** Surgical procedures.

Characteristics	Patients (*n* = 38)
Surgery type	
McKewon	16 (42.1%)
Ivor Lewis	22 (57.9%)
Number of total dissected LNs (range)	29 (9–58)
Operation time, median (range), min	315 (296–419)
Blood loss, median (range), mL	196 (134–431)
Hospital stays, median (range), days	16 (12–24)
Resection margins	
R0	36 (94.7%)
R1	2 (5.3%)
Postoperative complications	
Pneumonia	6 (15.8%)
Chylothorax	5 (13.2%)
Pleural effusion	4 (10.5%)
Wound infection	2 (5.3%)
Recurrent nerve paralysis	2 (5.3%)

### Clinical treatment response

The radiologic and pathologic responses are summarized in Table 4. Of all 38 patients, 3 (7.9%) patients had a complete response (CR), 21 (55.3%) patients had a partial response (PR), and 14 (36.9%) had a stable disease (SD). The objective response rate (ORR) was 63.2% and the disease control rate (DCR) was 100.0%. According to postoperative pathological results, 19 (50.0%) patients had a MPR, 15 (39.5%) patients had a pCR, and 4 (10.5%) patients were non-responders.

**Table 4:** Radiologic and pathologic responses.

Characteristics	Patients (*n* = 38)
Radiologic responses	
Complete response (CR)	3 (7.9%)
Partial response (PR)	21 (55.3%)
Stable disease (SD)	14 (36.9%)
Objective response rate (ORR)	24 (63.2%)
Disease control rate (DCR)	38 (100.0%)
Pathologic responses	
Non-responder	4 (10.5%)
Major pathological response (MPR)	19 (50.0%)
Complete pathological response (pCR)	15 (39.5%)

### Survival profiles

**Figure 1:**
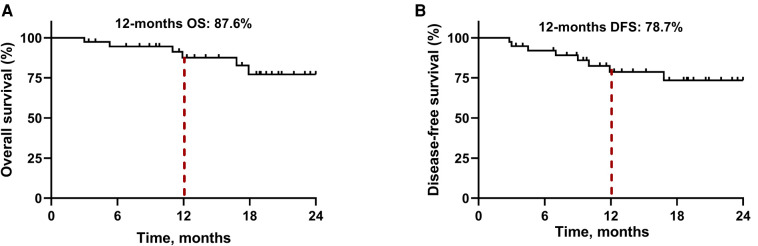
Kaplan-Meier survival curves in all patients. (**A**) Overall survival. (**B**) Disease-free survival.

**Figure 2:**
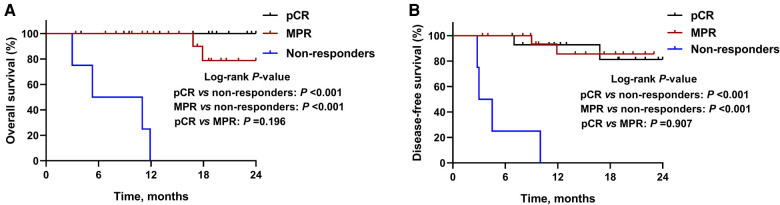
Kaplan-Meier survival curves stratified by pathological responses. (**A**) Overall survival and (**B**) disease-free survival for patients with major pathological response (MPR), complete pathological response (pCR), or non-responders.

**Figure 3:**
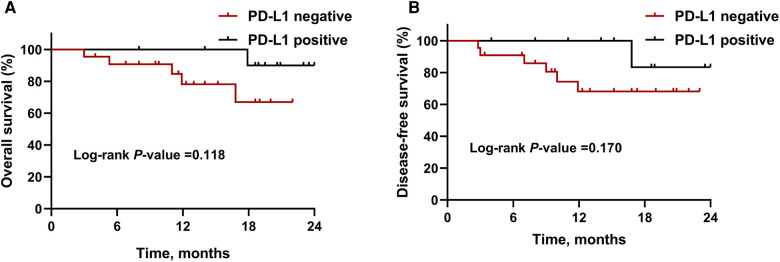
Kaplan-Meier survival curves stratified by PD-L1 expression. (**A**) Overall survival and (**B**) disease-free survival for patients with PD-L1 negative or positive expression. Abbreviations: PD-L1, programmed death-ligand 1.

By January 1, 2022, the median follow-up period was 18.5 months (range 3.4–28.5) and 6 (15.8%) patients had died. As demonstrated in Figure 1, the median OS and DFS was not reached in all patients; the OS rate was 87.6% at 12 months, 77.2% at 18 months, and 77.2% at 24 months; the DFS rate was 78.7% at 12 months, 73.5% at 18 months, and 73.5% at 24 months. In subgroup analyses, patients with a MPR or pCR achieved better OS (all *P *< 0.001, Figure 2A) and DFS (all *P *< 0.001, Figure 2B) than non-responders. Despite no significant statistical difference, patients with positive tumor PD-L1 expression tended to be associated with longer OS (*P* = 0.170, Figure 3A) and DFS (*P* = 0.118, Figure 3B).

## Discussion

This is a retrospective study to assess neoadjuvant camrelizumab plus chemoradiotherapy for locally advanced ESCC patients, which achieved a promising MPR rate of 50.0%, a pCR rate of 39.5%, and the R0 resection rate of 94.7%. This neoadjuvant therapy regimen showed optimal survival outcomes with an OS of 87.6% and a DFS of 78.7% at 12 months. This neoadjuvant treatment was well tolerated with a manageable safety profile.

Previous concern with neoadjuvant treatment remains the delay of surgery due to disease progression or serious TRAEs during neoadjuvant treatment. In our study, no patient progressed during the neoadjuvant treatment duration. This neoadjuvant treatment regimen resulted in no more than Grade 3 adverse events and the toxicity was manageable. All TRAEs were similar to either modality given alone in ESCC as previously reported (19–23). The incidence of radiation pneumonitis was relatively lower in our study compared with concurrent chemoradiotherapy (2.6% vs. 9.6–20.3% in grade 2; 0% vs. 1.8–7.4% in grade 3). Moreover, the incidence of radiation esophagitis did not increase compared with concurrent chemoradiotherapy (7.9% vs. 16.9–32.7% in grade 2; 7.9% vs. 3.2–5.6% in grade 3). Reactive capillary endothelial proliferation was the most common immune-related adverse event reported in camrelizumab monotherapy (75%–79%) (21, 24). The results of this study showed a much lower frequency (44.7%) of reactive capillary endothelial proliferation, and was all in grade 1 without special treatment. In addition, the surgical procedure was safe and controllable, as blood loss was minimal, operative time and hospital stay were reasonable, and the incidence of surgical complications was low. All the results indicated that the quality of our neoadjuvant treatment did not increase the risk of surgery and decrease the quality of surgery.

To data, several studies have evaluated the therapeutic efficacy of neoadjuvant PD-1blockade combined with chemotherapy or chemoradiotherapy for patients with ESCC. For instance, Yang et al. reported a pCR rate of 33.3% and a MPR of 41.7% in patients with locally advanced ESCC who have received neoadjuvant camrelizumab plus chemotherapy (12). Moreover, another retrospective study demonstrated that an R0 resection rate of 96.3%, a pCR rate of 33.3%, and an ORR of 88.9% were achieved in patients with locally advanced ESCC treating neoadjuvant nivolumab or pembrolizumab plus chemotherapy (13). In one retrospective study evaluating locally advanced ESCC patients receiving neoadjuvant immunotherapy (camrelizumab, pembrolizumab, or sintilimab) plus chemotherapy, the pCR rate was 34.21%, the MPR rate was 42.1%, and the R0 resection rate was 92.11% (14). In another study including 16 cases conducted by Yang et al., the ORR was 81.3%, the DCR was 100%, the pCR rate was 31.3%, and the R0 resection rate was 93.8% in locally advanced ESCC patients receiving neoadjuvant camrelizumab plus chemotherapy (15). They also reported the survival profile including a 1-year PFS of 83% and OS of 90.9%. In this study, 19 (50.0%) patients had a MPR, 15 (39.5%) patients had a pCR, and the survival was favorable with an OS of 87.6% and a DFS of 78.7% at 12 months. Overall, much more impressive results of pathological responses and survival outcomes were determined in the present study compared with the above studies. Moreover, this study adopted a rather homogenous PD-1 inhibitor regimen (camrelizumab) with a relatively larger number of patients.

Data of subgroup analysis demonstrated that patients who got MPR or pCR were associated with improved survival, which further reinforce the widely usage of pathological response as surrogate clinical endpoints for long-term survival (25–27). In this study, despite no statistically significant difference, patients with higher PD-L1 expression tended to have longer survival. PD-L1 expression remains the commonly explored biomarker for predicting the response to anti-PD1 therapy in several cancers including lung cancer (28), melanoma(29), and gastric cancer (30), whereas biomarker role of PD-L1 expression was disputable when analyzing the association between PD-L1 expression and the response to a PD-1 blockade (31). In ESCORT and ATTRACTION-3 studies, tumor PD-L1 expression was not a robust biomarker of the survival benefit for patients with advanced ESCC (7, 24). The correlation of PD-L1 expression and clinical outcomes are warranted for further investigation in ESCC.

There are sone limitations to this study. First, this is a retrospective study with inherent selection bias, which needs to be clarified in further prospective research. Second, the sample size of eligible patients in this study is relatively small. Third, the follow-up period was relatively short, and further survival analysis including 3- or 5-year survival rates should be performed in the future.

## Conclusions

Neoadjuvant camrelizumab plus chemoradiotherapy exhibits good feasibility and safety in treating patients with locally advanced ESCC. More prospective studies are needed to validate the expected efficacy of this neoadjuvant therapy.

## Data Availability

The original contributions presented in the study are included in the article/Supplementary Material, further inquiries can be directed to the corresponding author/s.
